# Cheap Advertising Dentists—Extraordinary Verdict

**Published:** 1851-01

**Authors:** 


*Cheap Advertising Dentists—Extraordinary Verdict.*—Nostrum venders with their empirical recipes of universal prescription, ushered into the world as they are by flashing papers, and many colored handbills, with counterfeit recommendations, followed though they be by the dead march to the graves with the groans of tortured thousands, are of the same grade with the class of practitioners above referred to. They are generally rejected *attachés* to the profession of medicine, whose slow and honorable industry lags too much on the road to fortune, to suit their abandoned rapacity for gold. Forcing themselves into its ranks by the use of such means as forcibly expels them, they rush into our miscellaneous world and belabor their willing victims, with millions of compound and simple pills, and wash them away in the tide of tonics and elixirs, which often, nay constantly, drench every community. And the chief virtue is that they they cure all for only two shillings.

The cheap advertising dentist does the same in a different way. He is often found driven from a more fitting employment in the capacity of a country blacksmith, or in the employment of some useful but less skillful of the mechanic arts, by sheer indolence and incapability, or more frequently by the inducement of an old sinner, who promises in the short space of thirty days, to qualify him for the practice of dental surgery, in all its branches. Thus we have those who migrate from the pocket of one community to that of another, depending only upon the *furor* they create, by flaming advertisements of cheap dentistry, being wholly and naturally incapacitated, even to approximation, to any qualification to practice.

Such we suppose to be the position held by the plaintiff, in the following singular and unjust suit: the verdict against the defendant is no less strange than deplorable.

From our European budget, it appears that a widow lady, had been attracted by the flaming advertisement of a dentist, who occasionally visited the place of her residence, and many other adjoining villages. In his advertisement, every possible success was promised with the most complete satisfaction to patients. After several visits, she arranged to have a set of teeth inserted, and in due time they were completed and placed in the mouth. Upon her return home she found all her hopes annihilated, the operation being a complete failure. Of course she again visited the person, who, upon examination, admitted the want of adaptation; accordingly a second set were made with a view of correcting the faults of the first. The second set however proved to be also a failure, with increased defects in construction. The price charged was twenty-five guineas for each set, which sums the defendant refusing to pay, the plaintiff brought an action for recovery of these amounts. In the course of trial, several dentists gave evidence pro and con. But a Mr. Rogers, as also a Mr. Hackley, both practitioners of eminence, pronounced a severe and entire condemnation on the pieces. The jury, notwithstanding, brought in a verdict of damages against the defendant for the full amount of both sets.

